# Efficient Use of Carbon Fibers as Heating Elements for Curing of Epoxy Matrix Composites

**DOI:** 10.3390/molecules26165095

**Published:** 2021-08-23

**Authors:** Lykourgos C. Kontaxis, Ioannis E. Chontzoglou, George C. Papanicolaou

**Affiliations:** Composite Materials Group, Department of Mechanical Engineering and Aeronautics, University of Patras, 26500 Patras, Greece; chontzoglou.j@gmail.com (I.E.C.); gpapan@upatras.gr (G.C.P.)

**Keywords:** epoxy resin curing, resistance heating, joule heating, carbon fibers, polymer–matrix composites (PMCs), three-point bending, flexural modulus, flexural strength

## Abstract

The aim of this study is to achieve a fully cured thermoset matrix that is heated by a direct electric current passing through the reinforcement fibers i.e., the Joule heating effect. Two types of fibers were used as heating elements for curing the epoxy resins. Kanthal resistance fibers were used as reference heating elements and subsequently, they were replaced by a Torayca Carbon Tow of the same radius. The specimens were cured by the heat produced by a direct electric current passing through the fibers and achieving temperatures of 50 °C and 70 °C. Specimens cured in a conventional oven were also manufactured, to compare the resistance heating method to the conventional one. Next, all specimens were mechanically characterized in a quasi-static three-point bending mode of loading and experimental results were compared to derive useful conclusions concerning the applicability of the technique to polymer/composite materials mass production. Finally, a preliminary economical study concerning power consumption needed for the application of both the traditional oven curing and the carbon fibers heating elements use for the manufacturing of the same amounts of materials is presented, showing a maximum financial benefit that can be achieved, on the order of 68%.

## 1. Introduction

To the present day, thermoset polymers and especially epoxy resins are used extensively for high-performance applications because of the increasing demand for lightweight and low-cost structural parts. Due to their strength-to-weight and high stiffness-to-weight ratio, as well as their excellent performance under fatigue and corrosion, fiber-reinforced polymer composite materials have demonstrated superior performance, in comparison to more conventional metallic materials, in several demanding aerospace, automotive, marine, wind energy, infrastructure, and consumer applications. The best example of the increasing use of advanced thermoset polymer composites is the Boeing 787 Dreamliner [1].

Although epoxy is a vital element both in the industrial and commercial world, it must first undergo a process of curing since epoxy only gets its practical use from creating a three-dimensional network of cross-links. It is generally accepted that uncured epoxy resins have poor mechanical, chemical, and heat resistance properties. However, the optimal above-mentioned properties are obtained by reacting the linear epoxy resin with suitable curing agents and then heating the compound, to form the three-dimensional cross-linked thermoset structures. This process is commonly referred to as the curing or gelation process [2]. Due to the formation of these structures, highly sought-after properties arise from these cured epoxy resins such as high modulus, high strength, low deformation, high glass transition temperature, low shrinkage, antifouling efficiency [3], and most importantly for composites; high adhesion to fillers and fiber reinforcement. However, their cross-linked structure leaves them with poor resistance to crack propagation [4].

The most widely used curing process for advanced thermoset polymer composites in industry is the oven or autoclave. In many cases, most commonly in the marine and aviation industry, an oven, or an autoclave suitable for a rather large part is not available or has limited availability. For example, “ASC Process Systems”, a company in the United States of America developed the largest autoclave (working area: ⌀ 9 m × 25 m) to satisfy the curing demands from the Boeing-787’s epoxy/carbon fiber composite parts. Nonetheless, the high financial cost and the size limitations of the autoclave ovens are still a great challenge to overcome, which is limiting the increased use of polymer composites in the wind and aerospace industry [5]. However, thermal curing in thermosets can be achieved using a wide range of thermal heating processes. Processes such as radiation curing, induction heating, convection and conduction heating ultrasonic heating, and resistance heating have been used for the curing of thermoset polymers, either in research or in industry [6].

The radiation curing method utilizes high energy electromagnetic radiation such as gamma-ray or X-ray [7,8,9], ultraviolet [10,11,12] or accelerated electron beams [9,13,14,15,16], and even visible light [17] for the ionization (bond breakage) of radiation-sensitive polymers since radiation curing is initiated by the ionic or free radical intermediates decomposed by the radiation sensitive resin on irradiation. On the contrary, thermal curing is by far the most popular curing method for polymer composites, with a great variety of thermal heating processes for thermal curing already in application. These processes can be categorized according to the heating mechanism present into radiation heating (infrared, laser, and microwave) [18], convection & conduction heating (hot gas, flame oven, and hot shoe) [19,20,21], induction heating [22,23,24,25,26], ultrasonic heating [27,28], thermal additive-based heating [29,30], and resistance heating [31].

Resistance heating, otherwise known as Joule heating or ohmic heating effect, describes the process where the energy of an electric current is converted into heat as it flows through a resistance. Concerning thermosets and/or thermoplastics, resistance heating is mostly utilized for composite bonding or welding processes, by using heating to heat up the interface and pressure to create bonds between materials [32,33,34,35,36,37]. Studies have shown that by directly applying an electric current, the electric conductivity of carbon fibers provides a potential to use them as a heating element [33,38]. However, according to Abliz et al. [6], due to the specific electric application nature of the heating process, it is mostly applicable for welding. To our knowledge, very little research work relating to curing of composite structures by this process is noticed to present, using this heating technology, which has been reported occasionally and in different expression forms, such as joule heating [39,40,41], resistance or resistive heating [42,43,44,45], embedded heating [46], and self-resistance electric (SRE) heating/curing [47]. These similar techniques have been applied by researchers, where the curing of carbon fiber reinforced polymer composite plates was achieved by the direct application of an electric current flown through the carbon fibers [39,41,42,47,48].

The aim of this study is to achieve a fully cured thermoset matrix that is heated by a direct electric current passing through the reinforcement fibers, i.e., the Joule heating effect. Two types of fibers were used as heating elements for the polymerization reaction to occur. At first, Kanthal resistance wires were used as reference heating elements, and, subsequently, they were replaced by a Torayca Carbon Tow of the same radius. The specimens were cured by the heat produced by an electric current passing through the fibers and achieving temperatures of 50 °C and 70 °C. The same procedure was applied to both the specimens with incorporated Kanthal and carbon fibers alike. For comparison reasons, with the conventional way of curing, specimens with fibers incorporated were also cured in an oven. Next, all specimens were mechanically characterized in a three-point bending mode of loading and experimental results were compared to derive useful conclusions concerning the applicability of the technique to polymer/composite materials mass production. Finally, a crude economical study concerning power consumption needed for the application of both the traditional oven curing and the carbon fibers heating elements use for the manufacturing of the same amounts of materials is presented.

## 2. Results and Discussion

In the present section, experimental findings regarding the flexural modulus and strength, of all types of specimens manufactured will be presented. They are divided according to the fiber heating element used as well as the number of fiber heating elements incorporated in each specimen (one or two). In addition, in each case (modulus or strength), cumulative charts concerning the curing method, or the fiber heating element choice are presented and discussed.

**Figure 1:**
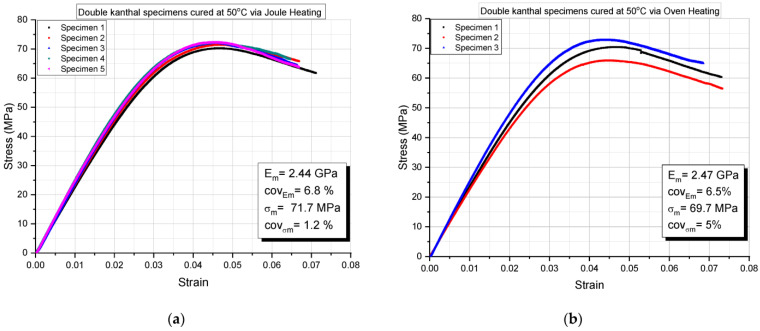
Quasistatic three-point bending results for double Kanthal fiber specimens cured at 50 °C using (**a**) Joule heating and (**b**) oven heating.

In order to show the repeatability of experimental results, in Figure 1, representative quasistatic three-point bending results for epoxy resin-double Kanthal fiber specimens cured at 50 °C are presented. More precisely, in Figure 1a epoxy specimens were cured via the resistance heating method, while in Figure 1b epoxy specimens were cured in an oven. From these two figures, and from the flexural modulus and flexural strength values derived from the stress-strain curves presented, the repeatability of experimental results can be observed. Flexural moduli for both curing methods are practically equal, and flexural strengths are almost equal as well. Both cases correspond to a 24 h curing time.

### 2.1. Flexural Modulus

**Figure 2:**
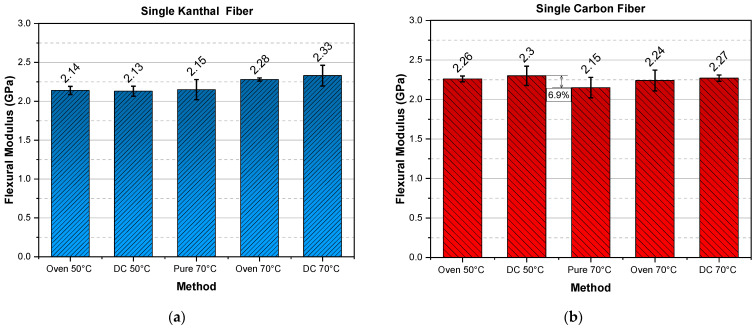
Flexural modulus versus curing method for (**a**) single Kanthal fiber specimens and (**b**) single carbon fiber specimens.

**Figure 3:**
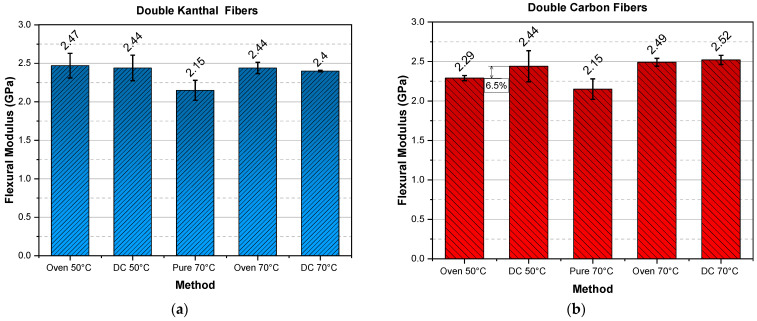
Flexural modulus versus curing method for (**a**) double Kanthal fiber specimens and (**b**) double carbon fiber specimens.

Since modulus is a sufficient first indicator of the successful curing of an epoxy resin, the mean values of the flexural moduli, for all types of specimens and curing procedures are shown in respective bar diagrams. Figures 2, 3, 5 and 6 are produced always following the same mode of presentation, i.e., the two bars, on the left side of each figure, are denoted as “Oven 50 °C” and “DC 50 °C”. This designation indicates that the specimens were cured at a temperature of 50 °C in an oven or via direct current (DC), respectively. In addition, specimens cured in an oven incorporated Kanthal or carbon fibers, to eliminate a variable that might affect the comparison of the two methods. Therefore, specimens without fibers, Kanthal or carbon, cured at a temperature of 70 °C were manufactured as a reference point. The middle bar in each of the aforementioned figures is dedicated to these specimens and is denoted as “Pure 70 °C”, meaning, it is a pure epoxy resin without any fiber heating element incorporated in it. Finally, the two bars on the right side of each figure, are denoted as “Oven 70 °C” and “DC 70 °C” and represent the specimens that were cured at a temperature of 70 °C in an oven or via direct current (DC), respectively. A 24 h curing time was chosen for all specimens and all cases to ensure that the epoxy resin was completely cured according to the literature [49,50,51,52].

To reiterate, the only specimens cured by Joule heating are the ones denoted with DC; the “Oven” specimens contain fibers but are cured in an oven, and the “Pure 70 °C” are cured in an oven and contain no fibers.

In Figure 2 the flexural moduli for single Kanthal fiber (Figure 2a) and single carbon fiber (Figure 2b) are presented. For single Kanthal fiber specimens the flexural moduli do not deviate depending on the curing method; for both temperatures (50 °C, 70 °C), flexural modulus remains almost constant, with a maximum variation of 8.3%, between pure oven cured and DC cured at 70 °C, epoxy polymers. The same conclusion can be drawn for the single carbon fiber specimens, however, the deviations between methods here are even smaller.

In Figure 3, the flexural moduli for double Kanthal fiber (Figure 3a) and double carbon fiber (Figure 3b) are presented. Except for the reference specimens (Pure 70 °C), the flexural moduli for double fiber specimens do not deviate depending on the curing method; for both temperatures (50 °C, 70 °C) and for both types of fibers (Kanthal, carbon), flexural modulus remains almost constant and around 2.45 GPa, apart from the case of double carbon fiber oven cured at 50 °C specimens (“Oven 50 °C”, Figure 3b). However, their deviation is on the order of 6.5% which is within the experimental error margins.

**Figure 4:**
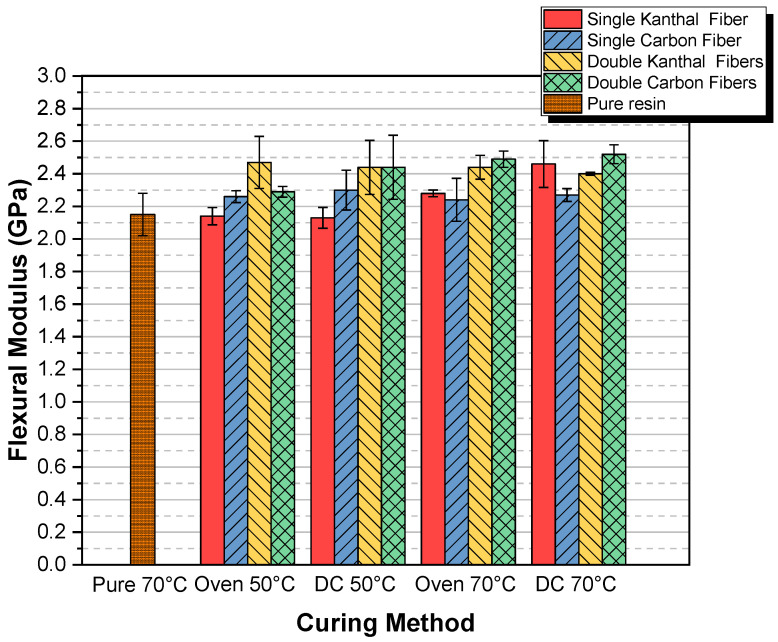
Flexural modulus versus curing method, for both types of fibers and for both single fiber and double fiber specimens.

Next, the cumulative diagrams for flexural modulus for all the above-presented cases are given. In Figure 4, the flexural modulus versus curing method, for both types of fibers and for both single and double fiber specimens, is given.

From Figure 4, we can conclude that the Pure 70 °C specimens showed the lowest flexural modulus. In addition, it becomes clear that independently of the method used, and for the same curing time of 24 h, the incorporation of fiber-heating elements into the epoxy leads to higher values in modulus. Finally, when comparing all methods to each other, it is obvious that the maximum flexural modulus achieved was that of the double carbon fibers DC 70 °C specimens showing a relative modulus enhancement with respect to pure 70 °C specimens on the order of 17%.

### 2.2. Flexural Strength

**Figure 5:**
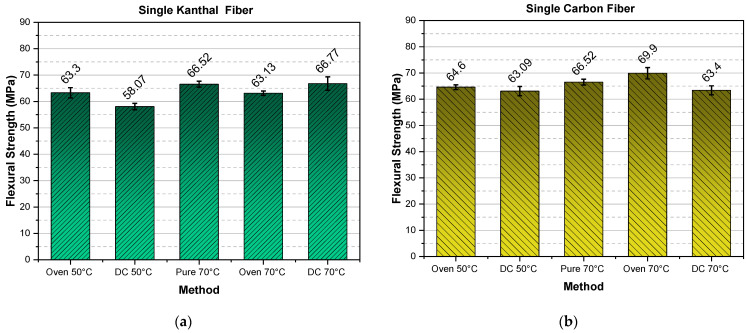
Flexural strength versus curing method for (**a**) single Kanthal fiber specimens and (**b**) single carbon fiber specimens.

**Figure 6:**
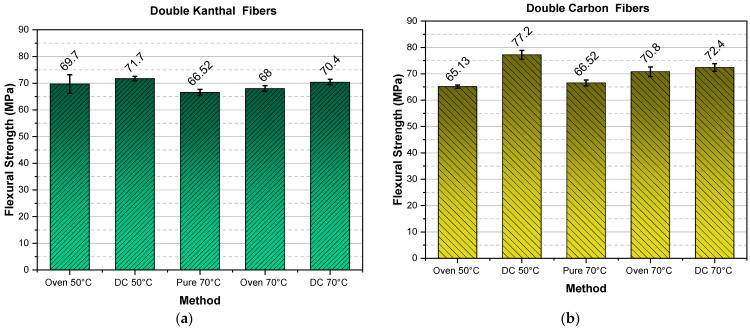
Flexural strength versus curing method for (**a**) double Kanthal fiber specimens and (**b**) double carbon fiber specimens.

In this sub-section, the mean values of the flexural strength, for all types of specimens and curing procedures are presented in the bar diagrams shown in Figure 5 and Figure 6. As already mentioned, the reference values corresponding to pure resin specimens cured in an oven, are always presented in the middle bar, and are denoted as “Pure 70 °C”.

For a better understanding of experimental results, results corresponding to single fiber heating elements are shown in Figure 5, while results referring to double fiber heating elements are presented in Figure 6.

It is well known that the introduction of fibers into an epoxy matrix leads to the creation of an interphase created in the area between fiber and matrix. This interphasial area is characterized by microcracks, impurities, voids, and localized concentration of stresses due to the fiber and matrix coefficient of thermal expansion discrepancies.

As a result of all the above-mentioned parameters, the specimen’s flexural strength is greatly affected, rendering this property unreliable for comparison, in relation to the respective modulus comparison. However, despite the above issues, as one can observe from Figure 5 and Figure 6, the double carbon fiber DC 50 °C and DC 70 °C give the highest strength values as compared with all the rest methods and fiber types. It must be stressed that all values presented correspond to 24 h curing time.

**Figure 7:**
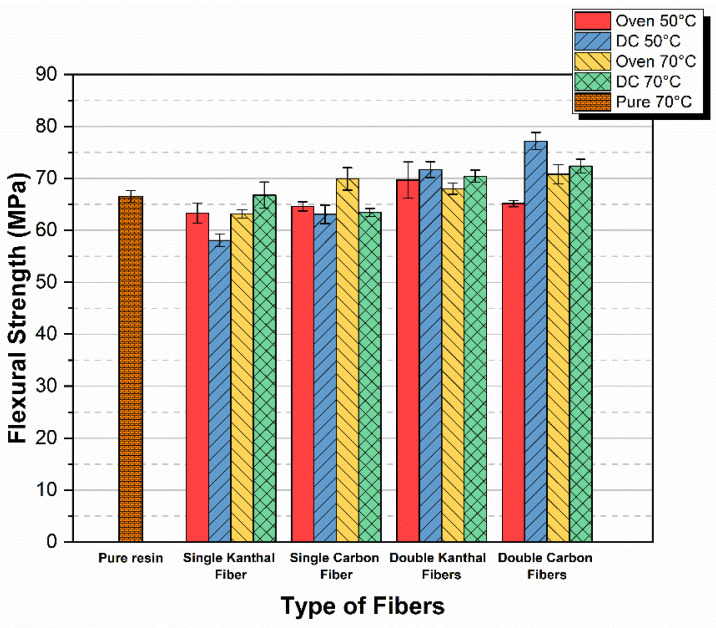
Flexural strength versus curing method, for both types of fibers and for both single fiber and double fiber specimens.

The superiority of the double carbon fiber heating element method is also verified in the bar diagram shown in Figure 7 where cumulative results already presented in Figure 5 and Figure 6 are given.

In Table 1, the flexural strength values presented in Figure 7 are supplemented with a statistical analysis of the results. A low standard deviation was found in all cases, indicating that the values tend to be close to the mean flexural strength values. Thus, the variation of the flexural strength values per case was very small and that is evident by the small coefficient of variation, with the largest being 5.01%.

Next, we calculated the mean flexural strengths for each type and number of fibers disregarding the curing method (Table 2) and also conducted a Weibull distribution statistical analysis.

It is evident by the statistical analysis presented in Table 2 that regardless of the method of curing (i.e., DC or Oven) and temperature (i.e., 50 °C or 70 °C), flexural strength does not variate significantly. The largest variation identified concerned the double carbon fiber specimens, and was on the order of 7%, while the smallest was exhibited by the double Kanthal fibers specimens, registering a meager 2.2% variation in flexural strength. This is attributed to the ductile nature of the material, which, unlike brittle materials, does not showcase a large variation in strength in general. It is also attributed to the complete polymerization of our epoxy resin after 24 h of curing, which normalized these values.

Finally, Weibull statistics for strength analysis was conducted. Weibull statistics is a well-established tool for the characterization of fracture strength of brittle materials [53], however, it can also be a valuable asset in determining the maximum strength of ductile materials. Weibull related the cumulative failure probability *P_f_* of a material with the stress *σ* using the following relationship

(1) $$P_{f}=1-exp\left[-{\left(\frac{\sigma -\sigma_{u}}{\sigma_{0}}\right)}^{m}\right],$$

where *m* is the Weibull modulus and *σ*_0_ is a scaling parameter. The *σ_u_* is the location parameter, denoting the stress at which there is a zero-failure probability; it is usually taken as zero for the safest assumption [54]. Equation (1) is known as the Weibull three parameter strength distribution. Setting *σ_u_* to zero in Equation (1) and taking the double logarithm of the resulting two-parameter Weibull distribution yields:

(2) $$P_{f}=1-exp\left[-{\left(\sigma /\sigma_{0}\right)}^{m}\right]$$

(3) $$\ln\left(1-P_{f}\right)=-{\left(\sigma /\sigma_{0}\right)}^{m}$$

(4) $$\ln\left[\ln\left(1/\left(1-P_{f}\right)\right)\right]=m\ln\sigma -{\mathrm{mln}\sigma }_{0}.$$

**Figure 8:**
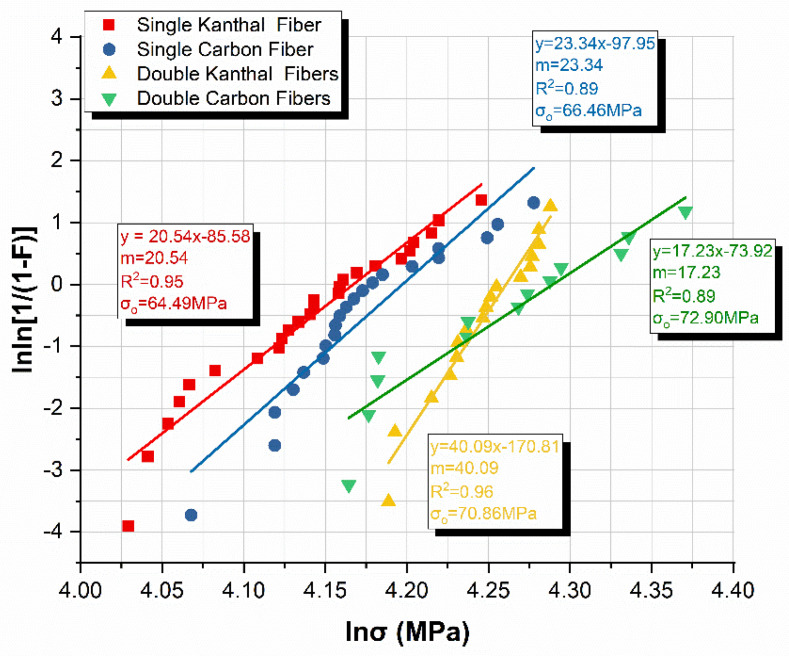
Weibull plot of flexural strength for each type and number of fibers.

For *N* nominally identical specimens ranked from the weakest (*i* = 1) to the strongest (*i* = *N*), the failure probability *P_f_* of the *i*_th_ one is calculated using the following equation

(5) $$P_{f,i}=\frac{n_{i}-0.5}{N},$$

where *n_i_* is the *i*th sample (*n_i_* = 1, …, *N* experiments) and *N* is the total number of samples tested. These results were then plotted in the usual double logarithmic form of the Weibull expression. Following the above-described procedure, Figure 8 was plotted for flexural strengths for each type and number of fibers disregarding the curing method. It can be seen from Figure 8 that the Weibull moduli (i.e., the slopes m) indicate the strength distribution widths. The similar slopes suggest that the same flaw types were active in most of the specimen sets. The not-so-linear curves are not unusual and are common in small-size sample sets. A high Weibull modulus, or steep slope, is associated with a narrow strength distribution. This is usually desirable, as materials with high Weibull moduli are more predictable and less likely to break at a stress much lower than a mean value [55]. The characteristic strength, or Weibull scale parameter *σ*_0_, is a location parameter that indicates the distribution location along the abscissa (x) axis; a large *σ*_0_ shifts the data to the right, while a small *σ*_0_ shifts the data to the left. The characteristic strength is the strength value, *σ*, at a failure probability *P_f_* = 63.2%, when *σ* = *σ*_0_ in Equation (2). Thus, reported Weibull characteristic strength values *σ*_0_ are slightly greater than the mean strength values (*P_f_* = 50%).

As expected from studying the coefficient of variations in Table 2 the double Kanthal specimens that exhibited the smallest variation (2.2%) have the steepest m slope, i.e., the largest Weibull modulus. On the contrary, the double carbon specimens have the smallest Weibull modulus and the largest variation (7%) as seen in Figure 8 and Table 2, respectively. Finally, the characteristic strength values *σ_0_* are marginally greater than the mean values reported in Table 2, as expected, thus validating the calculations of the statistical analysis.

### 2.3. DGEBA Verification Preliminary Study

**Figure 9:**
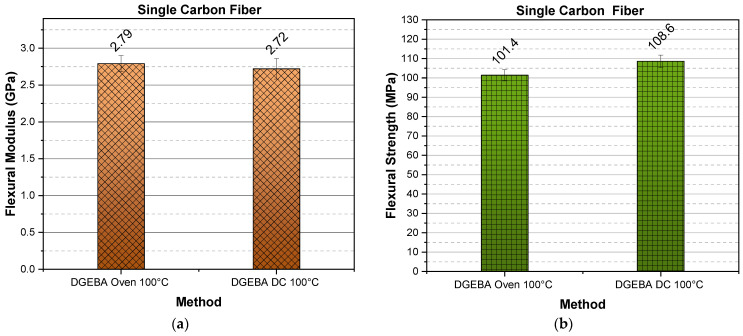
(**a**) Flexural modulus and (**b**) Flexural strength versus curing method for single carbon fiber specimens.

To verify the experimental results and confirm that this curing method is viable with other resins as well, an indicative preliminary study was conducted using epoxy resin DGEBA. In Figure 9, DGEBA’s flexural modulus and flexural strength are given for single carbon fiber heating element specimens, cured both in an oven and via resistance heating at 100 °C and for 24 h curing time.

As presented in Figure 9, the success of the method is once again verified. Both the oven cured, and the DC cured specimens, exhibit the same flexural modulus and strength values.

### 2.4. Economical Considerations

From an energy consumption point of view, a preliminary cost study was conducted, to determine which method is the most cost-effective. Therefore, the power consumed during the curing of the specimens in each case (i.e., oven, resistance heating) was estimated.

The oven used in the current study was the Binder GmbH type FD-35. The manufacturer’s datasheet provides the power consumption of the oven (Table 3).

Next, the energy consumption of the resistance heating method was calculated as follows:

For the Kanthal specimens, the resistance *R* was calculated by Pouillet’s law:

(6) $$R=\rho \frac{l}{A}.$$

where *ρ* is the resistivity of the material, *l* is the length, and *A* the cross-sectional area of the fiber.

The experimental resistance values and the calculated resistance values were nearly identical; therefore, the calculated values were used for the estimation of the power consumption using the equation

(7) $$P=\frac{V^{2}}{R},$$

where *P* is the Power consumption and *V* is the Voltage and assuming that the element behaves as a perfect resistor and that the power is completely converted into heat.

Concerning the carbon fibers used, the overall fiber is made up of 1000 microfibers of carbon, 7 μm in diameter each. The resistance of each individual microfiber is calculated from Equation (6). Assuming that the microfibers are connected in parallel, since they share the same voltage at their ends, by applying the relation

(8) $$\frac{1}{R_{tot}}=\sum_{i=0}^{1000}\left(\frac{1}{R_{i}}\right),$$

the nominal resistance of a 1 m carbon fiber is calculated to be *R_C tot_* = 441 Ω, while the multimeter confirmed these calculations with an experimental resistance of *R_C exp_* = 432 Ω. Again, the calculated values were used for the estimation of the power consumption of the carbon fiber specimens.

In Table 4, the power consumption of the specimens cured via Joule heating is presented. The calculated resistance and the voltage applied for each experimental setup are given, and the power consumption is calculated by Equation (7).

After calculating the power consumptions of both the conventional oven and the Joule heating curing method, the Energy consumption (kWh) for 24 h was calculated. In Greece, medium-sized industries use an industrial tariff with a fixed charge of 0.13 €/kWh. Thus, the cost of curing these specimens was calculated and presented in Table 5. In this table, the cost of eight molds manufactured was added since the oven used for this study has a maximum capacity of eight molds that can be cured simultaneously.

From the results presented in Table 5, it can be concluded that using carbon fiber as heating elements leads not only to superior mechanical properties, as already mentioned above, but also to financial benefits as well. From the data shown in Table 5, we can observe that the maximum financial benefit that can be achieved is on the order of 68%, corresponding to the case of the single carbon fiber heating element method. This is very important since, in carbon-fiber-reinforced polymers (CFRP) composites, the reinforcing carbon fibers themselves can be used as heating elements for the curing of the epoxy matrix, leading to products of the same or even better mechanical performance when compared to those cured with the traditional oven method, while at the same time led to financial benefits. At this point, we must stress that at an industrial level, and especially in the case of the huge number of prepreg lay-ups, the respective financial benefit would not be so high due to several technical and manufacturing complications. However, in any case, a financial benefit is expected to exist, although not at a level as high as that of 68%.

## 3. Materials and Methods

### 3.1. Materials

Two epoxy systems were utilized. The first and main one was an epoxy system encoded as RenLam CY219 (bisphenol A) combined with a curing agent HY 5161 (diamine) at a ratio of 2:1 by weight. The gelling time was 24 h at 50 °C and 70 °C, and the density of the cured polymer was 1.1 g·cm^−3^. The viscosity of the system CY219 and HY 5161 was 1–1.2 Pas at 25 °C. The second epoxy system used was D.E.R.332 (DGEBA) with a curing agent triethylenetetramine (TETA) at a ratio of 100:15 by weight. The felling time was 24 h at 100 °C, and the density of the cured polymer was 1.18 g·cm^−3^.

In addition, as already mentioned, two different types of heating elements were applied. The first one was that of Kanthal fibers, while the second one was that of carbon fibers.

Kanthal^®^ D is a ferritic iron-chromium (20.5–23.5%)-aluminum (4.8%) alloy (FeCrAl alloy) for use at temperatures up to 1300 °C. The alloy is characterized by high resistivity and good oxidation resistance.

The carbon fibers utilized in the present study were the PAN-based Torayca T300 1 k/66 tex (Toray Co., Tokyo, Japan). The name indicates that the overall fiber is made up of 1000 microfibers of carbon, ⌀ 7 μm each.

The properties of the Kanthal and carbon fibers used are given in Table 6.

### 3.2. Specimens Manufacturing

**Figure 10:**
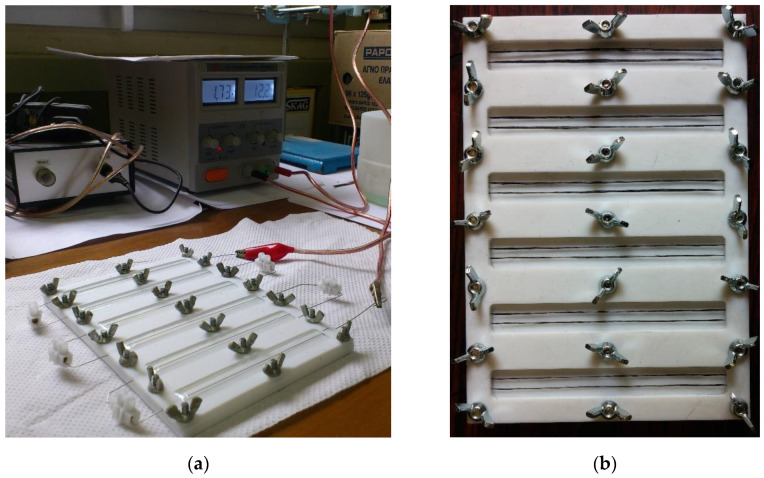
Experimental setup for (**a**) single Kanthal fiber specimens and (**b**) double carbon fiber specimens.

The molds used for the specimens manufacturing were made from Polytetrafluoroethylene (PTFE), commonly known as Teflon. This material was intentionally chosen due to its electrical insulation and non-stick properties. Initially, pure resin specimens were manufactured, which were cured in an oven, to establish a reference point. Then, resin specimens with a single Kanthal or carbon fiber incorporated were manufactured and an electric current was flown through the fibers to produce heat as a means of curing the resin (Joule Heating). In each case, the specimens were cured on two different temperature levels, 50 °C and 70 °C, with a curing time of 24 h. This was achieved by properly adjusting the voltage and measuring the temperature of the resistance medium by means of a thermocouple and a thermal camera. In addition, specimens with two Kanthal or carbon fibers per specimen were manufactured, to study the effect of the number of fiber heating elements on the curing of the resin. All tests were repeated; specimens containing either one or two fibers per specimen were cured in an oven in order to compare the proposed curing method with the traditional one. The experimental setup is shown in Figure 10.

### 3.3. Quasi-Static Mechanical Tests

The manufactured specimens underwent a series of quasi-static three-point bending tests (ASTM D790-03), by means of a universal mechanical testing machine Instron 4301(High Wycombe, UK). All tests were performed at room temperature to investigate the mechanical performance of the composites. In all cases, a constant crosshead speed of 1 mm/min was applied. All specimens had dimensions 100 × 12.8 × 3 mm and a span length of 63 mm. Five or more specimens per each case (i.e., oven cured or DC-cured) were tested to ensure the repeatability of results.

## 4. Conclusions

In the present investigation, single and double carbon and Kanthal fibers embedded in two different polymeric matrices were used as fiber-heating elements for the cure of the epoxy matrix. In addition, polymer specimens were cured following the traditional oven curing method for comparison. The results showed that carbon fibers, used in CFRPs as reinforcing elements, can also be used as heating elements for the epoxy curing procedure, leading to the same and or, in some cases, even better mechanical properties as compared to resins cured in an oven, at the same temperature and time. In addition, the use of carbon fibers heating elements leads to financial benefits which, in the case of a single carbon fiber heating element, was on the order of 68%.

## Figures and Tables

**Table 1:** Statistical analysis of flexural strength values for different curing methods.

Type of Fibers	Curing Method	Flexural Strength (MPa)	Standard Deviation (MPa)	Coeff. of Variation (%)
Pure Resin	Oven 70 °C	66.52	1.13	1.70
Single Kanthal Fiber	Oven 50 °C	63.30	1.94	3.06
	DC 50 °C	58.07	1.20	2.07
	Oven 70 °C	63.13	0.82	1.30
	DC 70 °C	66.77	2.54	3.80
Single Carbon Fiber	Oven 50 °C	64.60	0.88	1.36
	DC 50 °C	63.09	1.77	2.81
	Oven 70 °C	69.90	2.17	3.10
	DC 70 °C	63.41	0.77	1.21
Double Kanthal Fibers	Oven 50 °C	69.70	3.49	5.01
	DC 50 °C	71.72	1.53	2.13
	Oven 70 °C	68.03	1.09	1.60
	DC 70 °C	70.42	1.18	1.68
Double Carbon Fibers	Oven 50 °C	65.13	0.59	0.91
	DC 50 °C	77.18	1.67	2.16
	Oven 70 °C	70.8	1.84	2.60
	DC 70 °C	72.35	1.34	1.85

**Table 2:** Statistical analysis of flexural strength values for different type and number of fibers.

Type of Fibers	Flexural Strength (MPa)	Standard Deviation (MPa)	Coeff. of Variation (%)
Single Kanthal Fiber	62.81	3.58	5.70
Single Carbon Fiber	65.25	3.17	4.85
Double Kanthal Fibers	69.97	1.53	2.19
Double Carbon Fibers	71.37	4.97	6.98

**Table 3:** Power consumption of the Binder FD-35 oven.

Temperature (°C)	Power Consumption (W)
50	110
70	172
150	429
300	951

**Table 4:** Power consumption of Kanthal and carbon fiber specimens cured via Joule heating.

Materials	Number of Fibers	Temperature (°C)	Resistance (Ω)	Voltage (V)	Power Consumption (W)
Kanthal^®^ D	Single fiber	50	6.87	6	5.24
		70	6.87	8.4	10.27
	Double fiber	50	11.68	10.6	9.62
		70	11.68	15	19.30
Torayca T300 1 k/66 tex	Single fiber	50	441	44.1	4.41
		70	441	60	8.16
	Double fiber	50	441	44.1 ^1^	4.41 × 2 = 8.82 ^1^
		70	441	60 ^1^	8.16 × 2 = 16.32 ^1^

^1^ Due to the DC power supply’s inability to reach beyond 75 V half the double fiber carbon specimens were manufactured twice.

**Table 5:** Cost of curing and financial benefit.

Materials	Number of Fibers	Temperature (°C)	Power Consumption (W)	Energy Consumption (kWh)	One Mold Cost (€)	Eight Molds Cost (€)	Financial Benefit (%)
Kanthal^®^ D	Single fiber	50	5.24	0.126	0.0163	0.1308	61.89
		70	10.27	0.246	0.0320	0.2563	52.23
	Double fiber	50	9.62	0.231	0.0300	0.2401	30.04
		70	19.30	0.463	0.0602	0.4817	10.23
Torayca T300 1 k/66 tex	Single fiber	50	4.41	0.106	0.0138	0.1101	67.93
		70	8.16	0.196	0.0255	0.2038	62.03
	Double fiber	50	8.82	0.212	0.0275	0.2201	35.85
		70	16.32	0.392	0.0509	0.4075	24.06
Binder GmbH FD-35 oven	--	50	110	2.640	0.3432	0.3432	0.00
	--	70	172	4.128	0.5366	0.5366	0.00

**Table 6:** Heating elements properties.

Property	Kanthal^®^D	Torayca T300 1 k/66 Tex
Density [g/cm^3^]	7.25	1.76
Electrical Resistivity [Ω∙m]	1.35 × 10^−6^	17 × 10^−6^
Thermal Conductivity [W/m∙K]	11	10.46
Specific Heat [kJ/kg∙K]	0.46	0.79
Fiber Diameter ⌀ [mm]	0.5	7 × 10^−3^ per filament
Tensile Modulus [GPa]	220	230
Tensile Strength [MPa]	670 ^1^	3530

^1^ for 1 mm ⌀ fibers.

## Data Availability

Data supporting the reported results will be available with the corresponding author (L. C. Kontaxis).

## Sample Availability

Samples of the compounds are not available from the authors.
